# #MeToo – second wave a concern in health care, general practice included

**DOI:** 10.1080/02813432.2021.1880197

**Published:** 2021-03-15

**Authors:** Jørgen Nexøe

**Affiliations:** Research Unit of General Practice, University of Southern Denmark, Odense, Denmark

The #MeToo movement, which emerged into public consciousness and discourse in 2017, has been regarded as a historic milestone for those women who had been victims of sexual offending by men in positions of social influence.

Concerns of sexual harassment and violence towards women within the medical profession in general and within general practice and family medicine in particular, have also been raised in an editorial in this journal [1]. The Nordic Colleges of General Practitioners share a responsibility and were called for to launch and participate in a comprehensive program aiming at eliminating or at least minimizing the magnitude of this problem. Since, until now the rest is silence.

A second wave of the #MeToo campaign broke out in Denmark this autumn when the hostess of a Music award show, on stage told her story of sexual harassment from a superior during her junior employment in the Danish Broadcasting Corporation. Soon after that the leader of the Danish Social-Liberal Party had to resign because of admitted abusive behaviours. A few days later also the Lord Mayor of the city of Copenhagen had to resign because of accusations of sexual offending.

Also, within the medical profession the #MeToo movement has been revitalized lately. During September and October last year more than 1,000 predominantly female Danish doctors have signed a declaration against sexual offensive behaviour at work.

Participation in the movement by men who had been abused by women, has been noticeably absent until now. Sexual harassment is not just a feminist issue as some women also engage in sexual harassment although to a smaller extent than men. In Europe, it is estimated that one out of ten men, and approximately five out of ten women will experience some kind of sexual harassment during their career [2].

Lately, a number of Danish male doctors have come forward with stories of sexual harassments, unwanted sexual attention, and discrimination [3]. There is, however, no doubt that female doctors more frequently experience harassment – both from patients and colleagues [4].

Within general practice and family medicine, only few voices have been raised both during the first and second waves [1,5]. It is imperative to shed light on these issues both on a national level and on location within the individual general practice offices.

Are we ready to take action when we as GPs or practice nurses meet sexual harassment from patients and colleagues? We must ensure that the informal and cheerful communication in the clinic is perceived as such by all implicated, also by the resident. Are we capable to take care of colleagues and employed nurses when they have experienced sexual offensive behaviours from patients? What is the answer to the practice nurse who has objections against employing a definitely well qualified new male nurse colleague on the reason that he is most likely gay?

Sexual harassment and sexually offensive behaviours in the workplace are most often not a question of sex but rather a question of abuse of power and an unhealthy workplace culture.

As well as we have guidelines for treating diabetes and COPD we obviously need guidelines to handle sexual harassment and everyday sexism in an orderly manner [5]. This may be a way to avoid sexual violence and be a part of the obligation to secure good working environment for colleagues and employees.

It can be said that harsh tone of voice, bullying and sexually offensive acts are typically an inappropriate way of dealing with stress, insecurity and mistrust in work communities, which leads to one or more employees being offended, humiliated and ultimately excluded. It is therefore rooted in the culture of the workplace, which all employees help to create [6].

Everyone has a responsibility. If you behave passively, silently or neutrally, you are also helping to maintain a workplace culture that allows for such abusive behaviour.

The inappropriate workplace culture is a strain on the individual and can cause stress symptoms. At the same time, it creates insecurity throughout the work community and can impair the quality and efficiency of work. If the harsh tone of voice, bullying or sexually offending actions - for example wrapped in humour - are directed at colleagues, it can affect professionalism and ultimately patient safety.
